# Alkannin-Induced Oxidative DNA Damage Synergizes With PARP Inhibition to Cause Cancer-Specific Cytotoxicity

**DOI:** 10.3389/fphar.2020.610205

**Published:** 2020-12-22

**Authors:** Mingxin Chang, Hongge Wang, Jiajing Niu, Yan Song, Zhihua Zou

**Affiliations:** ^1^ Department of Gastrointestinal and Anal Surgery, China-Japan Union Hospital of Jilin University, Changchun, China; ^2^ Department of Cell Biology and Biophysics, School of Life Sciences, Jilin University, Changchun, China

**Keywords:** alkannin, oxidative DNA damage, PARP, DNA damage response, DNA repair, synergistic cytotoxicity

## Abstract

**Background:** Oncogenic transformation is associated with elevated oxidative stress that promotes tumor progression but also renders cancer cells vulnerable to further oxidative insult. Agents that stimulate ROS generation or suppress antioxidant systems can drive oxidative pressure to toxic levels selectively in tumor cells, resulting in oxidative DNA damage to endanger cancer cell survival. However, DNA damage response signaling protects cancer cells by activating DNA repair and genome maintenance mechanisms. In this study, we investigated the synergistic effects of combining the pro-oxidative natural naphthoquinone alkannin with inhibition of DNA repair by PARP inhibitors.

**Methods and Results:** The results showed that sublethal doses of alkannin induced ROS elevation and oxidative DNA damage in colorectal cancer but not normal colon epithelial cells. Blocking DNA repair with the PARP inhibitor olaparib markedly synergized with alkannin to yield synergistic cytotoxicity in colorectal cancer cells at nontoxic doses of both drugs. Synergy between alkannin and olaparib resulted from interrupted repair of alkannin-induced oxidative DNA damage and PARP-trapping, as it was significantly attenuated by NAC or by OGG1 inhibition and the non-trapping PARP inhibitor veliparib did not yield synergism. Mechanistically, the combination of alkannin and olaparib caused intense replication stress and DNA strand breaks in colorectal cancer cells, leading to apoptotic cancer cell death after G_2_ arrest. Consequently, coadministration of alkannin and olaparib induced significant regression of tumor xenografts *in vivo*, while each agent alone had no effect.

**Conclusion:** These studies clearly show that combining alkannin and olaparib can result in synergistic cancer cell lethality at nontoxic doses of the drugs. The combination exploits a cancer vulnerability driven by the intrinsic oxidative pressure in most cancer cells and hence provides a promising strategy to develop broad-spectrum anticancer therapeutics.

## Introduction

With the growing recognition that most malignantly transformed cells exhibit rewired metabolic pathways (Schulze and Harris, 2012; Boroughs and DeBerardinis, 2015; Liberti and Locasale, 2016), targeting cancer-specific metabolic vulnerabilities is currently being explored as a promising anticancer strategy (Luengo et al., 2017; Zecchini and Frezza, 2017; Wolpaw and Dang, 2018). A prominent metabolic feature shared by cancer cells is increased generation of reactive oxygen species (ROS) (Saikolappan et al., 2019). ROS are thought to be pro-tumorigenic, causing aberrant intracellular signaling that contributes to abnormal cell proliferation, metastasis, angiogenesis, and resistance to apoptosis (Irani et al., 1997; Reczek and Chandel, 2015). On the other hand, a large body of studies has demonstrated that cancer cells are vulnerable to further oxidative stress due to elevated intrinsic oxidative pressure, and agents that stimulate ROS generation or inhibit cellular antioxidant systems can drive cellular oxidative pressure to toxic levels selectively in tumor cells (Trachootham et al., 2006; Gorrini et al., 2013; Harris et al., 2015; AbdulSalam et al., 2016). Increased ROS can cause oxidative damage to nucleic acids (DNA and RNA), proteins and lipids (Zhang et al., 2011). Unrepaired oxidative DNA damage, such as DNA base lesions and single strand breaks (SSBs), may collide with replication forks during DNA repair and/or transcription leading to DNA replication stress and the formation of lethal DNA double-strand breaks (DSBs) to compromise cell survival (Markkanen, 2017; Srinivas et al., 2018). However, DNA damage response (DDR) signaling generally acts to protect cancer cells by regulating cell cycle checkpoints and activating DNA repair and genome maintenance activities (Ciccia and Elledge, 2010; Roos et al., 2016).

Poly(ADP-ribose) polymerase 1 and 2 (PARP1 and PARP2) play critical and overlapping roles in DNA repair and DDR (Ray Chaudhuri and Nussenzweig, 2017a). They are required for the repair of SSBs and a subset of DNA base lesions (Reynolds et al., 2015; Abbotts and Wilson, 2017; Ronson et al., 2018) and play regulatory roles in the repair of DSBs (Hanzlikova and Caldecott, 2019). Importantly, PARP1/2 are critical players in the stabilization and restart of stalled DNA replication forks (Yang et al., 2004; Bryant et al., 2009; Ray Chaudhuri and Nussenzweig, 2017a). Loss of both PARP1 and PARP2 activity is not lethal in adult cells with normal homology-directed repair (HDR) competency (Bryant et al., 2005; Farmer et al., 2005; Hanzlikova et al., 2017) but sensitizes cells to ionizing radiation and DNA-damaging genotoxic agents (Trucco et al., 1998; Menissier de Murcia et al., 2003). Moreover, cells lacking functional BRCA1 or BRCA2, hence deficient in HDR, are exquisitely sensitive to PARP inhibition (Bryant et al., 2005; Farmer et al., 2005). Thus, PARP inhibitors are used clinically to treat BRCA1/2 deficient breast and ovarian cancers, and they are effective and well tolerated (Lord and Ashworth, 2017). However, clinical use of PARP inhibitors in combination with radio- or chemotherapy to treat homologous recombination-proficient cancers has not been successful due to normal tissue toxicity (Yap et al., 2019). Given that DNA damage can be induced selectively in cancer cells by exogenous oxidative stress (Trachootham et al., 2006; Gorrini et al., 2013; Harris et al., 2015; AbdulSalam et al., 2016), it appears that combining pro-oxidative agents with PARP inhibition could be a promising approach to generate synergistic cancer lethality.

Alkannin and shikonin are enantiomeric natural naphthoquinones that have demonstrated broad-spectrum antitumor activity through diverse mechanisms (Papageorgiou et al., 1999; Zhang et al., 2018; Boulos et al., 2019; Pereyra et al., 2019; Wang et al., 2019). Like other natural and synthetic 1,4-naphthoquinones, alkannin and shikonin are able to undergo cyclic oxidation and reduction (redox cycling) that generates ROS and depletes antioxidants (Wu et al., 2005; Kumagai et al., 2012; Yang et al., 2014; Qiu et al., 2018). They also inhibit the thioredoxin reductase-1 (TrxR1) to cause accumulation of ROS (Duan et al., 2014) as well as the DNA topoisomerase I to induce DNA strand breaks (Plyta et al., 1998). Inhibition of ROS by the ROS inhibitor N-acetyl-cysteine (NAC) almost completely blocked the antitumor activity of alkannin and shikonin (Chang et al., 2010; Bogurcu et al., 2011; Gong and Li, 2011; Duan et al., 2014; Lee et al., 2014), suggesting that increased oxidative stress is the primary source of their cytotoxicity. Given the nonspecific nature of ROS toxicity and the diverse and pleiotropic bioactivities associated with naphthoquinones (Kumagai et al., 2012), it is difficult to achieve therapeutic effects without significant side effects using alkannin or shikonin as monotherapy (Lee et al., 2019; Wang et al., 2019). Similarly, although natural products have been a rich source of modern medicines and many anticancer drugs were derived from herbal or botanical preparations, a large number of traditional herbs possessing anticancer activities remain to be characterized, largely due to difficulties in defining their mechanisms of action (Li and Vederas, 2009; Kong et al., 2020). In this study, we show that nontoxic doses of alkannin induced oxidative DNA damage in colorectal cancer but not in normal colon epithelial cells. Sublethal alkannin-induced DNA damage sensitized colorectal cancer cells to the PARP inhibitor olaparib, and coadministration of alkannin and olaparib resulted in synergistic lethality in colorectal cancer cells and effectively suppressed the growth of tumor xenografts *in vivo*. These studies support further exploration of the synergistic cytotoxicity between PARP inhibitors and specific pro-oxidative agents to exploit a cancer vulnerability common to most oncogenically transformed cells.

## Materials and Methods

### Materials

The SW480 and SW1116 cell lines were purchased from the American Type Culture Collection (ATCC) (Manassas, VA, United States), and all other cell lines were bought from the Cell Bank of the Chinese Academy of Sciences (Shanghai, China). Cell authenticity was confirmed by short tandem repeats (STR) profiling.

Alkannin (B50783) was bought from Shanghai Yuanye Biotechnology (Shanghai, China). Olaparib (S1060), niraparib (S2741), talazoparib (S7048) and veliparib (S-1004) were purchased from Selleck (Houston, TX, United States). The OGG1 inhibitor O8 (SML1697) and N-acetyl-L-cysteine (NAC) (BP907) were purchased from Sigma-Aldrich (St. Louis, MO, United States). Stock solutions of alkannin, olaparib or veliparib were made in 100% dimethyl sulfoxide (DMSO) (Sigma-Aldrich) and working solutions were prepared in complete cell culture medium. Vehicle controls were prepared similarly but without the test compound. Primary antibodies include γH2AX-pS139 polyclonal antibody (ab11174) (Abcam, Cambridge, United Kingdom); cleaved caspase-3 (9664S), Chk1-pS317 (12302S) and Chk2-pT68 (2661S) (Cell Signaling, Danvers, MA, United States); Chk1 (bs1681R), GAPDH (bs2188R), β-actin (bsm33036M), CDC25C (bs10579R), CDC25C-pS216 (bs3096R), p53-pS15 (bs3702R), p53 (bs2092R), and PARP1 (bs2138R) (Bioss, Beijing, China); H2AX (abs131731), Chk2 (abs131635) and BBC3 (PUMA) (abs131259) (Absin Bioscience, Shanghai, China); 53BP1 (A300-272A) (Bethyl, Montgomery, TX, USA); γH2AX-pS139 monoclonal antibody (14-9865-82) (ThermoFisher, Waltham, MA, USA). Secondary antibodies include goat anti-mouse-Alexa 488 (115-545-003) and goat anti-rabbit-Cy3 (111-165-003) (Jackson ImmunoResearch, West Grove, PA, USA); goat anti-mouse-HRP (bs-40296G-HRP) and goat anti-rabbit-HRP (bs-40295G-HRP) (Bioss).

### MTT Assay

Cells were seeded in 96-well plates at 4 × 10^3^ cells/well for 12 h and were then treated with drugs for the indicated times. When multiple drugs were used, they were added simultaneously. At the end of drug treatment, 20 μL of 5 mg/ml 3-(4,5-dimethylthiazol-2-yl)-2,5-diphenyl tetrazolium bromide (MTT) (Sigma-Aldrich) was added to each well and incubated at 37 °C for 4 h. The media were carefully removed and 100 μL of DMSO was added to each well. The plate was left on a plate shaker for 30 min with gentle shaking at room temperature. Absorbance at 595 nm was measured. IC_50_ values were determined by the GraphPad Prism software using nonlinear regression analysis. The combination index (CI) values were determined by the Chou-Talalay method according to the formula CI = (D)1/(Dx)1 + (D)2/(Dx)2. (Dx)1 and (Dx2) were concentrations of each drug alone to exert x% effect, while (D)1 and (D)2 were concentrations of drugs in combination to elicit the same effect. CI < 1 indicated synergism, and CI = 1 or >1 indicated additivity or antagonism, respectively (Chou, 2010, 2018).

### Colony Formation Assay

Cells were seeded in 12-well plates at 500 cells/well for 12 h and were then treated with the indicated drugs for 7 days. Following washing with PBS, the cells were fixed in ice-cold methanol and briefly stained with crystal violet solution (0.5% crystal violet in 25% methanol) (Sigma-Aldrich). The plates were air-dried, images were taken, and the violet crystals were dissolved in 70% ethanol. Absorbance at 595 nm was measured.

### Measurement of Cellular ROS and MMP

Cells were seeded in 6-well plates at 1 × 10^5^ cells per well for 12 h and were then treated with drugs for the indicated times. Cellular ROS were detected using a cell-based ROS assay kit (Beyotime, Shanghai, China). Briefly, cells were washed with PBS, and incubated with 10 μM dichlorofluorescin diacetate (DCFH-DA) for 30 min at 37 °C in the dark. After washing three times in PBS, images were taken immediately, and the cells were then collected through trypsinization and analyzed on the BD FACS-Calibur flow cytometer (BD Biosciences, San Jose, CA, United States). Cellular ROS levels were expressed as the average DCF (di-chloro-fluorescin) intensity. Mitochondrial membrane potential (MMP) was measured using a commercial Mitochondrial Membrane Potential Assay kit with JC-1 following the manufacturer’s instructions (Beyotime). After drug treatment, cells were washed by PBS and incubated in JC-1 dye at 37 °C in the dark for 20 min, followed by washing twice with JC-1 buffer. Cells were covered by a thin layer of DMEM media for photographing.

### Immunofluorescent Staining

Cells on round coverslips or cryosections of tumor tissues were fixed in 4% paraformaldehyde (PFA) for 30 min and washed three times with PBS. For 8-oxoG staining, fixed cells were incubated in Cy3-conjugated avidin (Struthers et al., 1998) (Rockland Immunochemicals, Limerick, PA, United States) (0.5 μg/ml) for 1 h at room temperature; for immunofluorescent staining, the samples were incubated sequentially in blocking buffer (3% fetal bovine serum, 0.1% Triton X-100 in PBS), primary and secondary antibodies, each for 1 h at room temperature; for TUNEL assay, tissue sections were incubated in working solutions from a One-Step TUNEL apoptosis assay kit (Beyotime) for 60 min at 37 °C. Samples were then washed three times in PBS and sealed on glass slides in the VECTASHIELD Mounting Medium with DAPI (Vector Laboratories, Burlingame, CA, United States).

### Flow Cytometry

Cells were seeded in 6-well plates at 1 × 10^5^ cells per well, treated with drugs for the lengths of time indicated in the figure legends, and collected through trypsinization. The cell pellets were washed twice in PBS before the following analyses. For cell cycle analysis, cell pellets were fixed in 70% ice-cold ethanol at −20 °C for 1 h. After washing in PBS twice again, cells were stained with working solutions from the Cell Cycle Detection kit (Bestbio, Shanghai, China). DNA content was revealed by propidium iodide. For the analysis of apoptosis, cells were resuspended in a binding buffer from the Annexin V-FITC Apoptosis Detection kit (Bestbio) according to the instructions provided by the manufacturer. The samples were analyzed by the MoFlo XDP Cell Sorter (Beckman Coulter, Indianapolis, IN, United States). 2 × 10^4^ cells were analyzed per sample.

### Alkaline Comet Assay

Measurement of DNA strand breaks in individual cells by the alkaline comet assay (single cell gel electrophoresis assay) was performed according to the instructions included in the OxiSelect Comet Assay kit (Cell Biolabs, San Diego, CA, United States). After drug treatment, cells grown in 6-well plates were collected through trypsinization. Cell pellets were resuspended in 1.2% low-melting point agarose maintained at 37 °C at 10 × 10^5^ cells/ml, which were then layered on a frosted slide from the OxiSelect Comet Assay kit. The slides were stored at 4 °C overnight in pre-cooled lysis buffer (100 mM EDTA, 2.5 M NaCl, 10 mM Tris–HCl, 1% Triton X-100 and 10% DMSO, pH 10.0). After washing twice with an enzyme buffer (40 mM HEPES, 0.1 M KCl, 0.5 mM EDTA and 0.2 mg/ml BSA, pH 8.0), the slides were denatured in pre-chilled alkali buffer (300 mM NaOH, 1 mM EDTA) in a horizontal electrophoresis chamber for 30 min. Electrophoresis was then proceeded at 20 V and 300 mA in the same buffer for 30 min. After incubation in cold neutralizing buffer (250 mM Tris–HCl, pH 7.5) for 30 min, slides were immersed in cold 70% ethanol for 5 min and allowed to air dry. At the end, cells were stained with Vista Green DNA dye provided by the kit at room temperature for 15 min. Images were quantified using the free TriTek CometScore software (TriTek Corp., Sumerduck, VA, United States). At least 100 comets per sample were analyzed. Tail moment was determined as the percentage of DNA in the tail multiplied by the tail length.

### Western Blot

Cells grown in 6-well plates were scraped off the plates in 100 μL of radioimmunoprecipitation buffer (150 mM NaCl, 1.0% IGEPAL CA-630, 0.5% sodium deoxycholate, 0.1% sodium dodecyl sulfate, and 50 mM Tris, pH 8.0) (Sigma) with 1 mM phenylmethane sulfonylfluoride (Sigma). Samples were centrifuged at 4 °C for 20 min at 12,000 g and protein concentrations were determined by a BCA Protein Assay kit (Dingguo, Changchun, China). Proteins were denatured at 95 °C for 10 min, separated on a 12% SDS-PAGE gel, and transferred to PVDF membranes. The membranes were blocked in 5% (w/v) non-fat milk in TBST (10 mM Tris, 100 mM NaCl, 0.1% Tween 20, pH 7.5) for 1 h at room temperature, and then probed in specific first and second antibodies. Signals were developed using a Tanon-5200 chemiluminescence image analysis system (Tanon, shanghai, China).

### Tumor Xenograft Study

All animal studies were conducted in compliance with animal protocols approved by the Institutional Animal Care and Use Committee of Jilin University. 2 × 10^6^ SW480 cells were inoculated subcutaneously into the flank of 8-week old male athymic BALB/c nude mice (Charles River, Boston, MA, United States), and the mice were randomly placed in control and treatment groups (5 animals per group). Fifteen days after inoculation, tumors reached about 150 mm^3^ and the mice were treated once daily with 20 mg/kg alkannin oral gavage (p.o.) or 50 mg/kg olaparib intraperitoneal injection (i.p.), or a combination of alkannin and olaparib, for 15 days. Tumor volume was measured every 3 days, and tumor weight was measured at the end of treatment.

### Statistical Analysis

All *in vitro* experimental data were expressed as mean ± standard deviation (SD) of three independent experiments. All statistical comparisons were completed by the two-tailed Student’s *t*-test using the GraphPad Prism software (GraphPad Software, Inc., La Jolla, CA, United States). *p* < 0.05 was considered as statistically significant.

## Results

### Sublethal Doses of Alkannin Increase ROS Levels in Colorectal Cancer Cells

**FIGURE 1:**
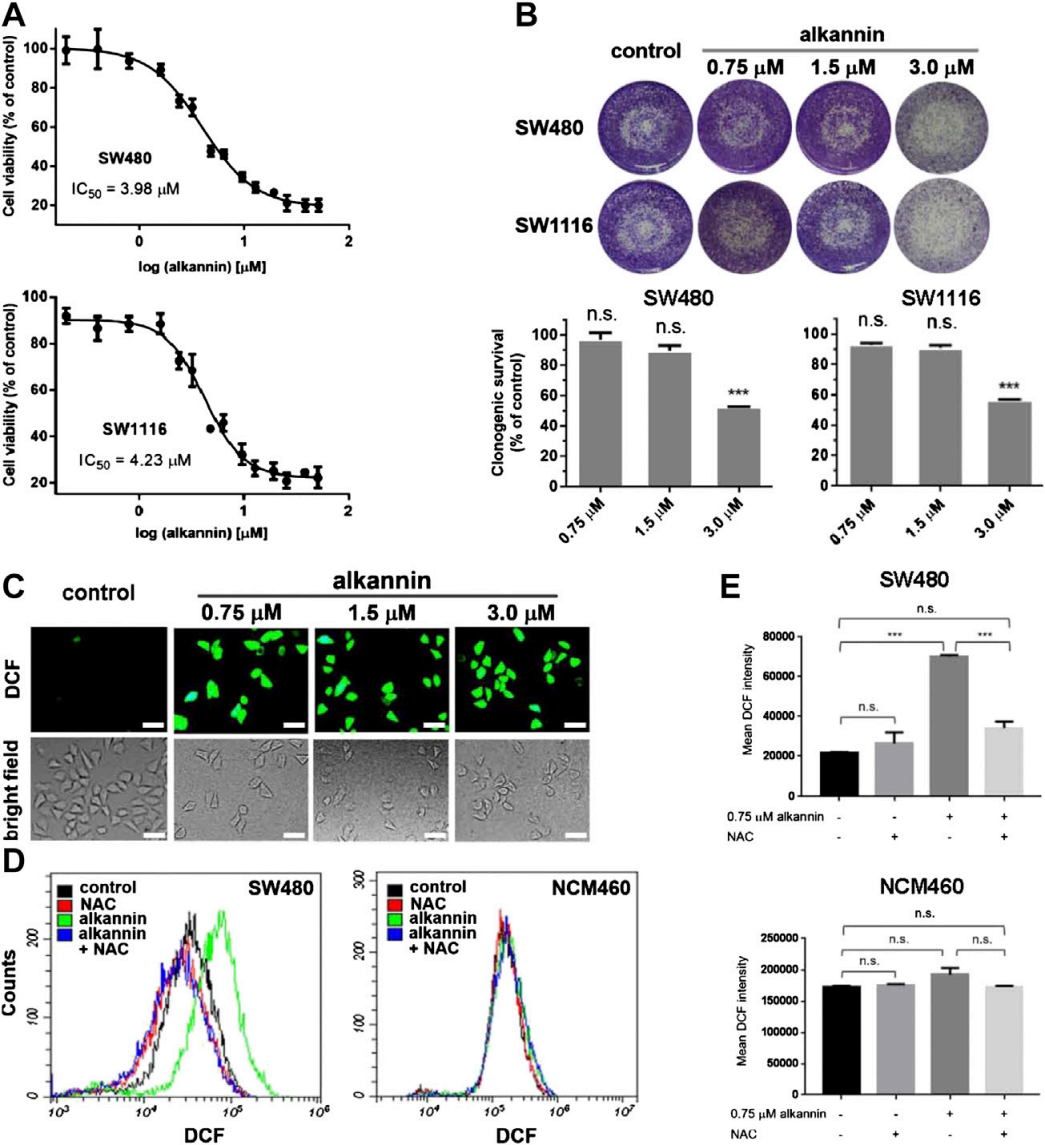
Sublethal doses of alkannin elevate ROS levels in colorectal cancer cells. **(A)** MTT assay. Cells were treated with 0.1, 0.2, 0.4, 0.8, 1.6, 2.4, 3.2, 4.8, 6.4, 9.6, 12.8, 19.2, 25.6, 38.4 or 51.2 μM alkannin for 24 h. The IC_50_ values of alkannin against the SW480 and SW1116 colorectal cancer cell lines were calculated using the GraphPad Prism software. Data were shown as average ± SD from three independent experiments. **(B)** Colony formation assay. SW480 and SW1116 cancer cells were treated with alkannin at the indicated doses for 7 days and data from three independent experiments were presented as mean ± SD. **(C)** Representative images of DCFH-DA staining. SW480 cells were treated by alkannin at the indicated doses for 3 h (scale bars: 50 μm). **(D)** Measurement of ROS by flow cytometry. Treatment by 0.75 μM alkannin for 3 h induced a significant ROS increase in the SW480 cancer but not the NCM460 noncancerous cells. NAC suppressed the ROS increase in the cancer cells. **(E)** Quantification of flow cytometry measurements of ROS (*n* = 3). n.s.: not significant, *:*p* < 0.05, ***:*p* < 0.001.

Antitumor activity of alkannin was initially demonstrated in a mass screening of the US National Cancer Institute’s natural and synthetic compound repository in 1974 (Driscoll et al., 1974). Since then, a large body of studies has confirmed the cytotoxicity of alkannin against diverse types of cancer cell lines with IC_50_ values in the low micromolar range (1–30 μM) (Huang et al., 2004; Wu et al., 2005; Bogurcu et al., 2011). In agreement with the literature, we found that alkannin dose-dependently inhibited the growth of a panel of human solid tumor cell lines (Supplementary Figure S1A). After 24 h of treatment, the IC_50_ values of alkannin against the SW480 and SW1116 human colorectal cancer cell lines were 3.98 and 4.23 μM respectively (Figure 1A). Accordingly, 3 μM alkannin significantly suppressed the clonogenic growth of these two cell lines, but 1.5 and 0.75 μM alkannin had no impact on their clonogenic survival (Figure 1B). Nevertheless, all three doses of alkannin induced a marked increase in ROS levels in both cancer cell lines (Figure 1C; Supplementary Figure S1B).

Flow cytometry analyses revealed that, treatment with 0.75 μM alkannin for 3 h caused a significant increase in ROS levels in both SW480 and SW1116 cancer cells (Figures 1D–E, Supplementary Figure S1C). In contrast, similar treatment caused no change in ROS levels in the NCM460 normal human colon epithelial cell line (Figure 1D–E). The ROS inhibitor N-acetyl-L-cysteine (NAC) effectively reduced the alkannin-induced ROS increase in the cancer cells (Figures 1D–E; Supplementary Figure S1C). Together, these results showed that significant increases in ROS levels were induced in the colorectal cancer but not in the noncancerous colon epithelial cells by nontoxic doses of alkannin.

### Alkannin-Induced ROS Elevation Leads to Oxidative DNA Damage

**FIGURE 2:**
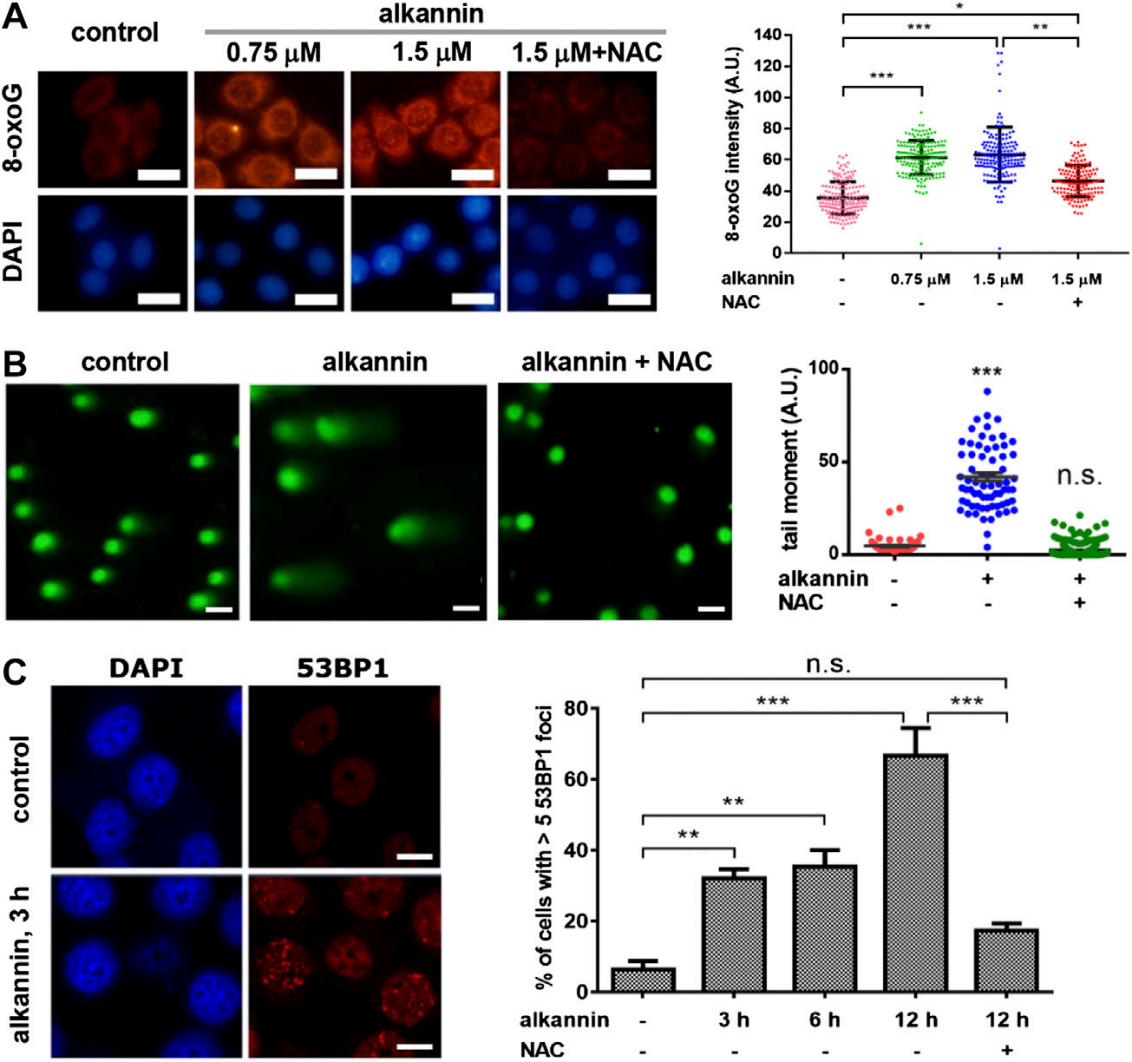
Sublethal alkannin-induced ROS elevation causes oxidative DNA damage. **(A)** Staining and measurement of cellular 8-oxoG. SW480 cells were treated by alkannin for 3 h and stained with Cy3-avidin (scale bars: 25 μm). Data from three independent experiments were presented as mean ± SD. **(B)** Alkaline comet assay. SW480 cells were treated by 0.75 μM alkannin for 3 h (scale bars: 25 μm). Tail moment was defined as percentage of tail DNA × tail length and was quantified using the TriTek CometScore software (*n* = 3). **(C)** 53BP1 staining. SW480 cells were treated by 0.75 μM alkannin for the indicated times (scale bars: 10 μm). 53BP1 positive cells were quantified using the ImageJ software (*n* = 3). n.s.: not significant, *:*p* < 0.05, **:*p* < 0.01, ***:*p* < 0.001.

Elevated ROS can cause oxidative DNA damage including nucleobase oxidization and single-strand DNA breaks (SSB); double-strand DNA breaks (DSB) may arise when a DNA replication fork collides with a SSB during DNA replication. Consistent with the increase in ROS levels, staining of 8-oxoguanine (8-oxoG), the major type of oxidized nucleobases (Markkanen, 2017), showed that within 3 h of treatment, both 1.5 and 0.75 μM alkannin induced a significant increase in 8-oxoG levels in the SW480 and SW1116 cancer but not in the NCM460 noncancerous cells (Figure 2A; Supplementary Figure S2A). NAC suppressed 8-oxoG increase in the cancer cells (Figure 2A; Supplementary Figure S2A), correlating 8-oxoG elevation with sublethal alkannin-induced ROS overload.

Similarly, alkaline comet assay revealed that 3 h of treatment by 0.75 μM alkannin caused a significant increase in DNA strand breaks in the SW480 and SW1116 cancer cells (Figure 2B; Supplementary Figure S2B). Moreover, 53BP1 foci, which represent sites of DSB (Schultz et al., 2000), were induced in the two colorectal cancer cell lines by 0.75 μM alkannin in a time-dependent manner (Figure 2C, Supplementary Figure S2C), suggesting that some of the DNA strand breaks detected by the alkaline comet assay were DSBs. No increase in DNA strand breaks was induced in the noncancerous NCM460 colon epithelial cells (Supplementary Figures S2B,C), and both the comet tails and 53BP1 foci in the cancer cells were effectively reversed by NAC (Figures 2B,C; Supplementary Figure S2C), indicating that the DNA strand breaks resulted from sublethal alkannin-induced ROS elevation.

### Sublethal Alkannin Sensitizes Colorectal Cancer Cells to Olaparib

**FIGURE 3:**
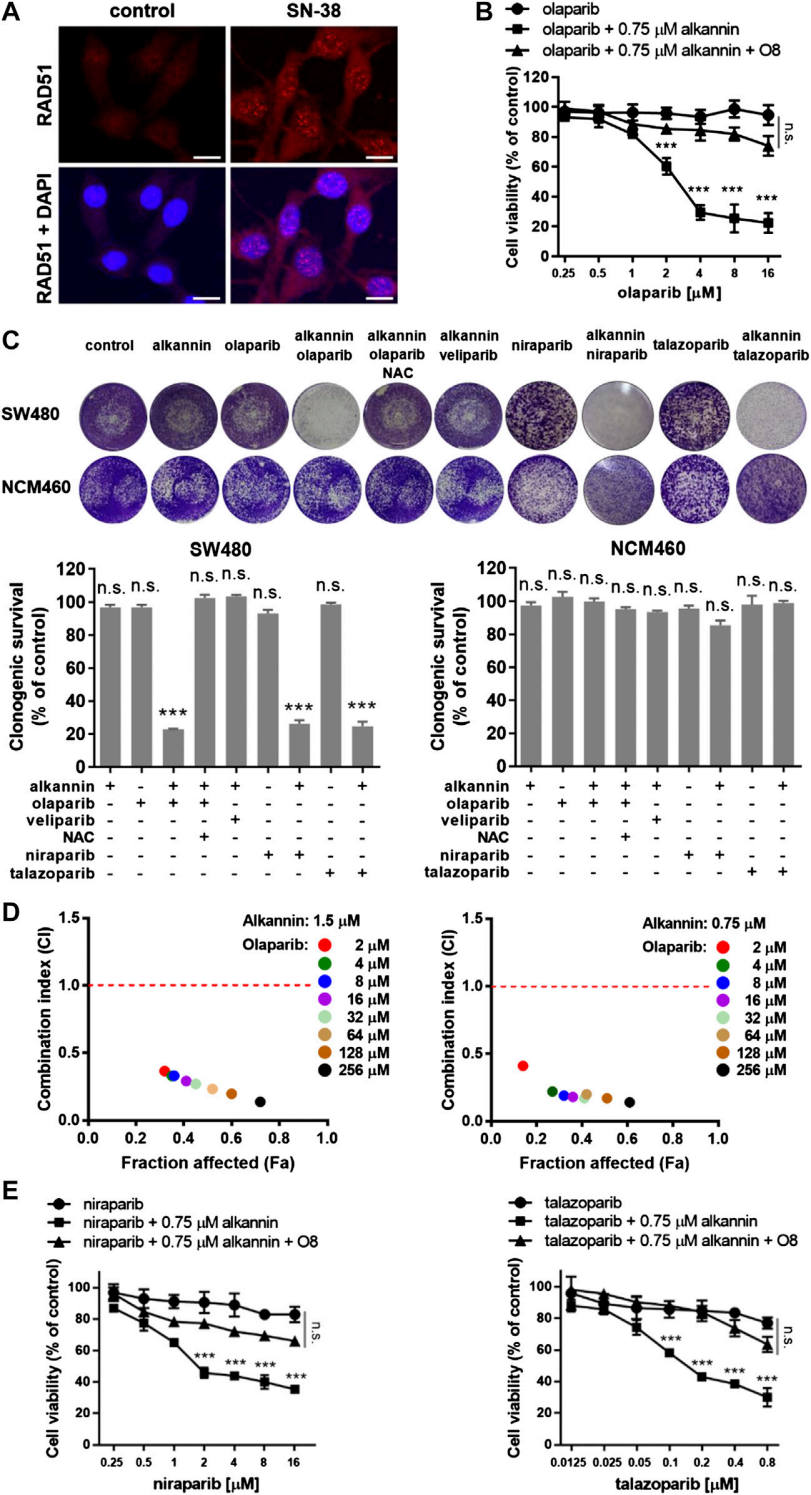
Sublethal alkannin sensitizes colorectal cancer cells to PARP-trapping. **(A)** SW480 cells readily formed RAD51 foci in response to 12 h of treatment by 5 μM SN-38, indicating that they were able to assemble recombination filaments normally (scale bars: 10 μm). **(B)** MTT assay. SW480 cells were treated with 0.125, 0.25, 0.5, 1.0, 2.0, 4.0, 8.0 or 16.0 μM olaparib alone or together with 0.75 μM alkannin with or without the OGG1 inhibitor O8 for 72 h. **(C)** Colony formation assay. SW480 and NCM460 cells were treated by the indicated drugs for 7 days (alkannin 0.75 μM, olaparib 10 μM, veliparib 10 μM, niraparib 5 μM, talazoparib 0.25 μM). Data from three independent experiments were presented as mean ± SD. **(D)** Determination of CI values. SW480 cells were treated by 1.5 or 0.75 μM alkannin combined with olaparib at the indicated concentrations (2, 4, 8, 16, 32, 64, 128 and 256 μM) for 72 h. **(E,F)** MTT assay. SW480 cells were treated with niraparib **(E)** or talazoparib **(F)** alone or together with 0.75 μM alkannin with or without the OGG1 inhibitor O8 for 72 h n.s.: not significant, ***:*p* < 0.001.

The induction of oxidative DNA damage led us to ask if nontoxic doses of alkannin could sensitize colorectal cancer cells to inhibition of PARP1/2. Homologous recombination (HR)-deficient cancer cells are exquisitely sensitive to PARP inhibition even in the absence of exogenous DNA damage. However, most types of cancer cells are HR-proficient and are thus resistant to PARP inhibition. Indeed, the SW480 and SW1116 cells readily formed RAD51 foci in response to treatment by 5 μM SN-38 (Figure 3A, Supplementary Figure S3A), indicating normal HR function (Graeser et al., 2010). Consistently, both cell lines were highly resistant to the PARP inhibitor olaparib (Figure 3B, Supplementary Figure S3B).

Remarkably, however, in the presence of 0.75 μM alkannin, olaparib dose-dependently inhibited the viability of the SW480 and SW1116 cancer cells (Figure 3B, Supplementary Figure S3B). Similarly, colony formation assays showed that neither 0.75 μM alkannin nor 10 μM olaparib had any impact on the clonogenic growth of the SW480 and SW1116 cancer cells but strikingly, the combination of 0.75 μM alkannin and 10 μM olaparib almost completely inhibited their clonogenic survival (Figure 3C, Supplementary Figure S3C). In contrast, the clonogenic growth of the NCM460 normal colon epithelial cells was not affected by the combination of 0.75 μM alkannin and 10 μM olaparib (Figure 3C). Thus, sublethal alkannin selectively sensitized the SW480 and SW1116 cancer cells to olaparib to yield synergistic cancer cell toxicity at nontoxic doses of both drugs.

To evaluate the synergism between alkannin and olaparib, the combination index (CI) was determined by the Chou-Talalay method using the CompuSyn software. The CI values for combinations between either 1.5 or 0.75 μM alkannin and a wide range of olaparib concentrations were far below 0.5, indicating strong synergy between alkannin and olaparib (Figure 3D, Supplementary Figure S3D).

### Synergy between Alkannin and Olaparib Results From Interrupted Repair of Oxidative DNA Lesions and PARP-Trapping

The synergistic cytotoxicity between alkannin and olaparib was greatly attenuated by NAC (Figure 3C, Supplementary Figure S3C), suggesting that it was dependent on alkannin-induced oxidative DNA damage. The 8-oxoguanine glycosylase (OGG1) initiates base excision repair (BER) of 8-oxoG by excising oxidized guanin bases and further cleaving the DNA backbone, leading to generation of SSB. PARP1/2 bind SSBs produced directly or as intermediates of BER, and recruit XRCC1 to assemble the repair machinery (Ray Chaudhuri and Nussenzweig, 2017b). Thus, inhibition of PARP would lead to accumulation of SSBs, while inhibition of OGG1 would reduce the generation of SSB and mitigate the impact of PARP inhibition. Consistently, the OGG1 inhibitor O8 (Donley et al., 2015) greatly reduced the cytotoxicity of the alkannin and olaparib combination (Figure 3B, Supplementary Figure S3B), suggesting that OGG1-initiated base excision repair of alkannin-induced 8-oxoG was a major cellular activity that synergized with olaparib to generate synergistic cytotoxicity.

In addition to inhibiting the activity of PARP1/2, many PARP inhibitors, such as olaparib, rucaparib, niraparib and talazoparib also trap the PARP protein at DNA damage sites to generate additional cytotoxicity (Murai et al., 2012; Lord and Ashworth, 2017; Murai and Pommier, 2019). In contrast, veliparib is much weaker at trapping PARP1/2 on damaged DNA and is therefore considered as a PARP inhibitor with no PARP-trapping potency. Interestingly, the combination of 0.75 μM alkannin and 10 μM veliparib produced no impact on the clonogenic growth of the SW480 and SW1116 cancer cells (Figure 3C, Supplementary Figure S3C), suggesting that veliparib did not synergize with the sublethal alkannin to generate synergistic cytotoxicity. On the contrary, both niraparib and talazoparib, two PARP inhibitors with higher PARP-trapping potency than olaparib, dose-dependently inhibited the viability of the SW480 and SW1116 cancer cells in the presence of 0.75 μM alkannin (Figures 3E,F, Supplementary Figures S3E,F). The OGG1 inhibitor O8 also greatly reduced the cytotoxicity of the alkannin and niraparib or alkannin and talazoparib combination (Figures 3E,F, Supplementary Figures S3E,F). Similarly, the combination of 0.75 μM alkannin and 5 μM niraparib, or 0.75 μM alkannin and 0.25 μM talazoparib, completely inhibited the clonogenic survival of the SW480 and SW1116 cancer cells (Figure 3C, Supplementary Figure S3C). Thus, PARP-trapping was likely the major cause of the synergistic cytotoxicity between alkannin and PARP inhibitors with PARP-trapping potency.

### Alkannin and Olaparib together Induce Intense Replication Stress and Extensive DNA Breaks

**FIGURE 4:**
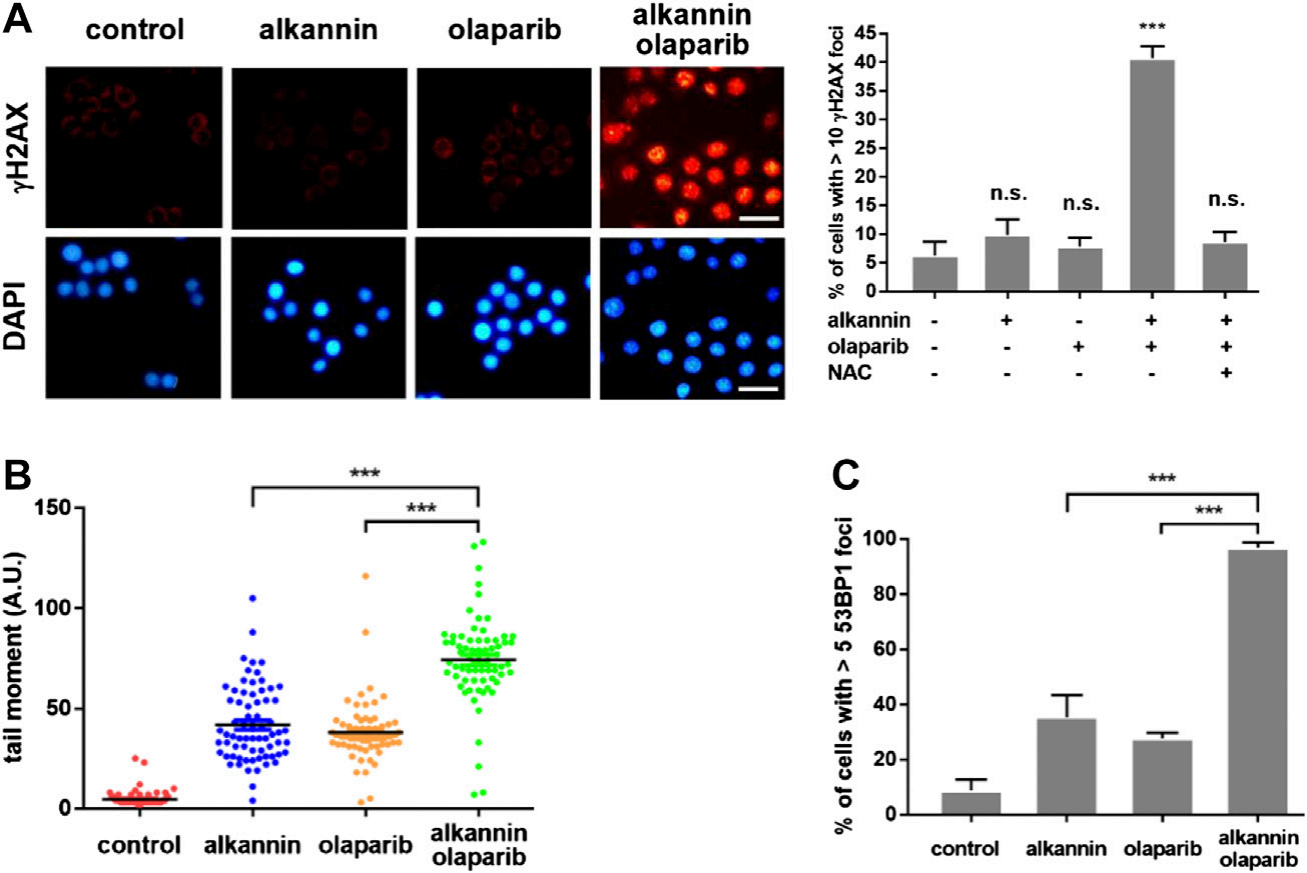
The combination of alkannin and olaparib induces intense replication stress and extensive DNA strand breaks. SW480 cells were treated by 0.75 μM alkannin, 10 μM olaparib or the combination of the two, with or without NAC, for 3 h. **(A)** Immunofluorescent staining of γH2AX (scale bars: 25 μm). Data from three independent experiments were presented as mean + SD. n.s.: not significant, ***:*p* < 0.001 vs vehicle control. **(B)** Alkaline comet assay and **(C)** 53BP1 staining (*n* = 3). ***:*p* < 0.001, alkannin and olaparib combined vs alkannin or olaparib alone.

Inhibition of PARP1/2 leads to accumulation of SSBs. In addition, PARP inhibitors with PARP-trapping property produce trapped PARP-DNA complexes which, together with unrepaired SSBs, collide with DNA replication forks, leading to fork stalling and replication stress (Pommier et al., 2016; Ray Chaudhuri and Nussenzweig, 2017b). Staining of γH2AX, a molecular marker of DNA replication stress and/or DSBs (Ward and Chen, 2001), revealed that 3 h of treatment by the combination of 0.75 μM alkannin and 10 μM olaparib, but not by each agent alone, caused a marked increase in the number of γH2AX positive SW480 and SW1116 cells, most of which displayed strong pan-nuclear γH2AX staining (Figure 4A, Supplementary Figure S4A), indicating induction of intense replication stress (Moeglin et al., 2019) specifically by the combination of alkannin and olaparib. The staining of γH2AX was reversed by NAC (Figure 4A, Supplementary Figure S4A), correlating the intense replication stress with sublethal alkannin-induced oxidative DNA damage.

Because PARP1 and PARP2 also play critical roles in the stabilization and restart of stalled DNA replication forks, PARP inhibition can impair replication fork stabilization and restart, which, together with the replication stress simultaneously induced by PARP inhibition, will promote replication fork collapse and generation of one-ended DSBs (Hanzlikova and Caldecott, 2019). Consistent with the specific induction of strong replication stress, alkaline comet assays revealed that the combination of alkannin and olaparib induced significantly higher levels of DNA strand breaks than the individual drugs (Figure 4B, Supplementary Figure S4B). Similarly, 53BP1 staining showed that significantly more 53BP1 positive SW480 and SW1116 cells were induced by the combination than by each agent alone (Figure 4C, Supplementary Figure S4C), suggesting that the intense replication stress induced by the combination of alkannin and olaparib was converted to DSBs.

### Activation of DNA Damage Response Leads to G_2_ Arrest and Apoptosis

**FIGURE 5:**
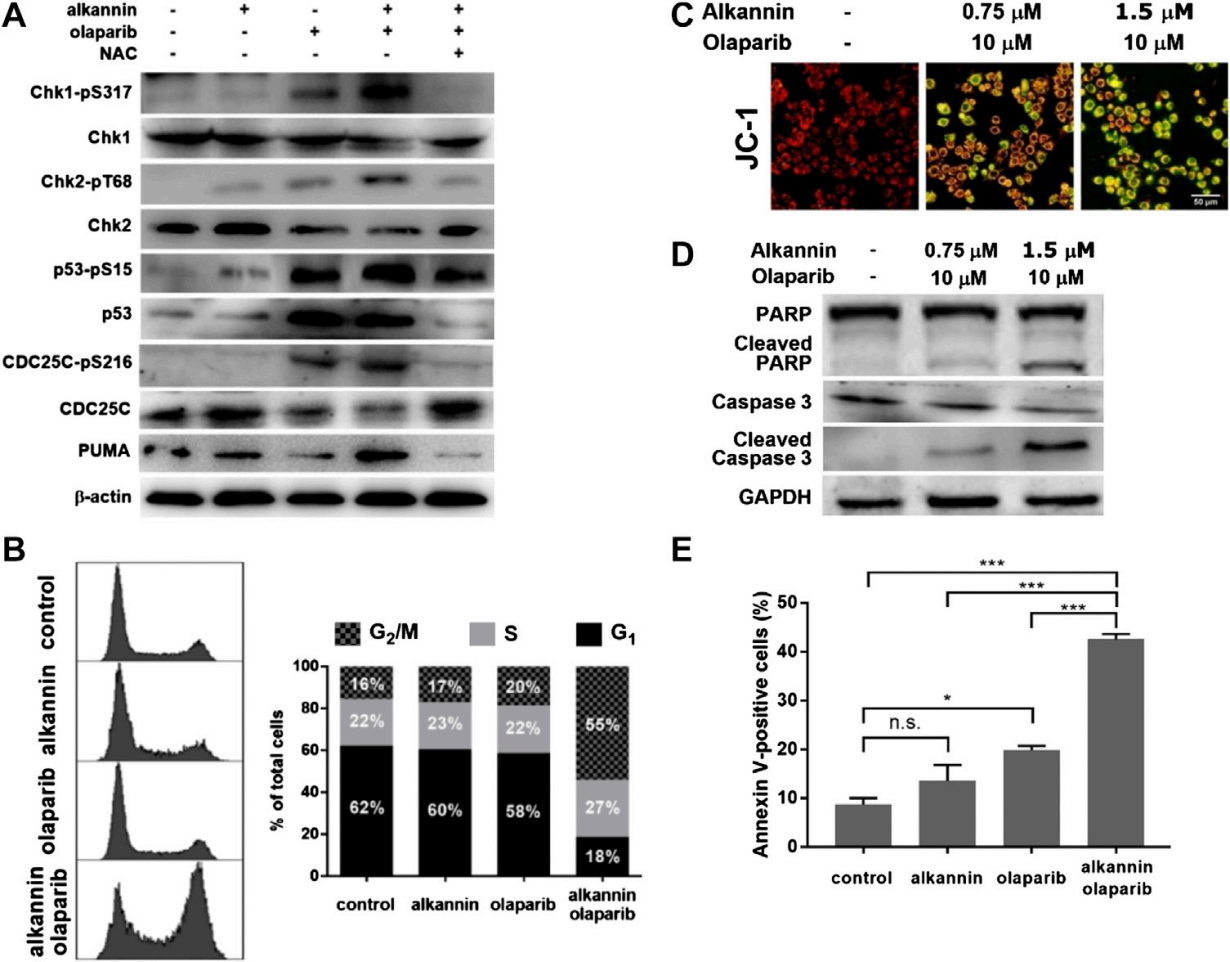
DDR activation leads to cell cycle arrest and apoptosis. **(A)** Western blot. SW480 cells were treated by 0.75 μM alkannin, 10 μM olaparib or the combination of the two, with or without NAC, for 3 h. **(B)** Flow cytometry analysis of cell cycle. SW480 cells were treated by 0.75 μM alkannin, 10 μM olaparib or the combination of the two for 48 h (*n* = 3) (note: the subG_1_ population was excluded from these measurements). **(C)** Measurement of mitochondrial membrane potential and **(D)** Western blot. SW480 cells were treated by the combination of 1.5 or 0.75 μM alkannin and 10 μM olaparib for 12 h. **(E)** Flow cytometry analysis of apoptosis. SW480 cells were treated by 0.75 μM alkannin, 10 μM olaparib or the combination of the two for 48 h (*n* = 3). n.s.: not significant, *:*p* < 0.05, ***:*p* < 0.001.

In response to replication stress and/or DSB, DDR is activated to regulate cell cycle checkpoints and DNA repair that maintain cell viability; or to induce programmed cell death that eliminates the cells with irreparable problems. In the SW480 and SW1116 cancer cells, 3 h of treatment by the combination of 0.75 μM alkannin and 10 μM olaparib induced a marked increase in the levels of phosphorylated Chk1, Chk2 and p53 (Figure 5A, Supplementary Figure S5A), demonstrating activation of both the ATM-Chk2 and the ATR-Chk1 DDR pathways (Ciccia and Elledge, 2010). The levels of phosphorylated Chk1, Chk2 and p53 were notably reduced by NAC (Figure 5A, Supplementary Figure S5A), correlating DDR activation with the specific induction of intense replication stress and extensive DNA strand breaks by the combination of alkannin and olaparib. A weaker increase in the levels of phosphorylated Chk1, Chk2 and p53, as well as a significant increase in total p53, was induced by olaparib alone (Figure 5A), indicating that blocking PARP1/2 activity alone was able to activate DDR but could not produce cytotoxicity.

In accordance with DDR activation, phosphorylation of CDC25C, one of the important downstream targets of DDR, was significantly increased, which was accompanied with a corresponding significant decrease in the levels of the total CDC25C protein (Figure 5A, Supplementary Figure S5A). Consistently, flow cytometry analyses showed that, 48 h of treatment by the combination of 0.75 μM alkannin and 10 μM olaparib, but not by each alone, induced a dramatic decrease in the number of cells in G_1_ and a marked increase in the number of cells in G_2_/M (Figure 5B, Supplementary Figure S5B), indicating thorough activation of the G_2_/M cell cycle checkpoint specifically by the combination of alkannin and olaparib.

Importantly, the proapoptotic BH3-only protein PUMA, a downstream target of p53 and direct activator of apoptosis, was significantly upregulated specifically by the combination of alkannin and olaparib (Figure 5A, Supplementary Figure S5A), consistent with that the SW480 and SW1116 colorectal cancer cells have normal p53 function (Rochette et al., 2005; Leroy et al., 2014). Correspondingly, staining with the fluorescent probe JC-1 showed a profound dissipation of mitochondrial membrane potential (Figure 5C, Supplementary Figure S5C), and Western blot analyses revealed a significant increase in the levels of activated caspase 3 and cleaved PARP (Figure 5D, Supplementary Figure S5D), in the SW480 and SW1116 cancer cells treated by the combination of 1.5 or 0.75 μM alkannin and 10 μM olaparib for 12 h. Furthermore, flow cytometry analysis showed that 48 h of treatment by the combination of 0.75 μM alkannin and 10 μM olaparib, but not by each of them alone, induced a marked increase in the percentage of Annexin V-positive cells (Figure 5E, Supplementary Figure S5E), demonstrating full activation of the mitochondrial apoptotic pathway.

### The Combination of Alkannin and Olaparib Induces Regression of Tumor Xenografts

**FIGURE 6:**
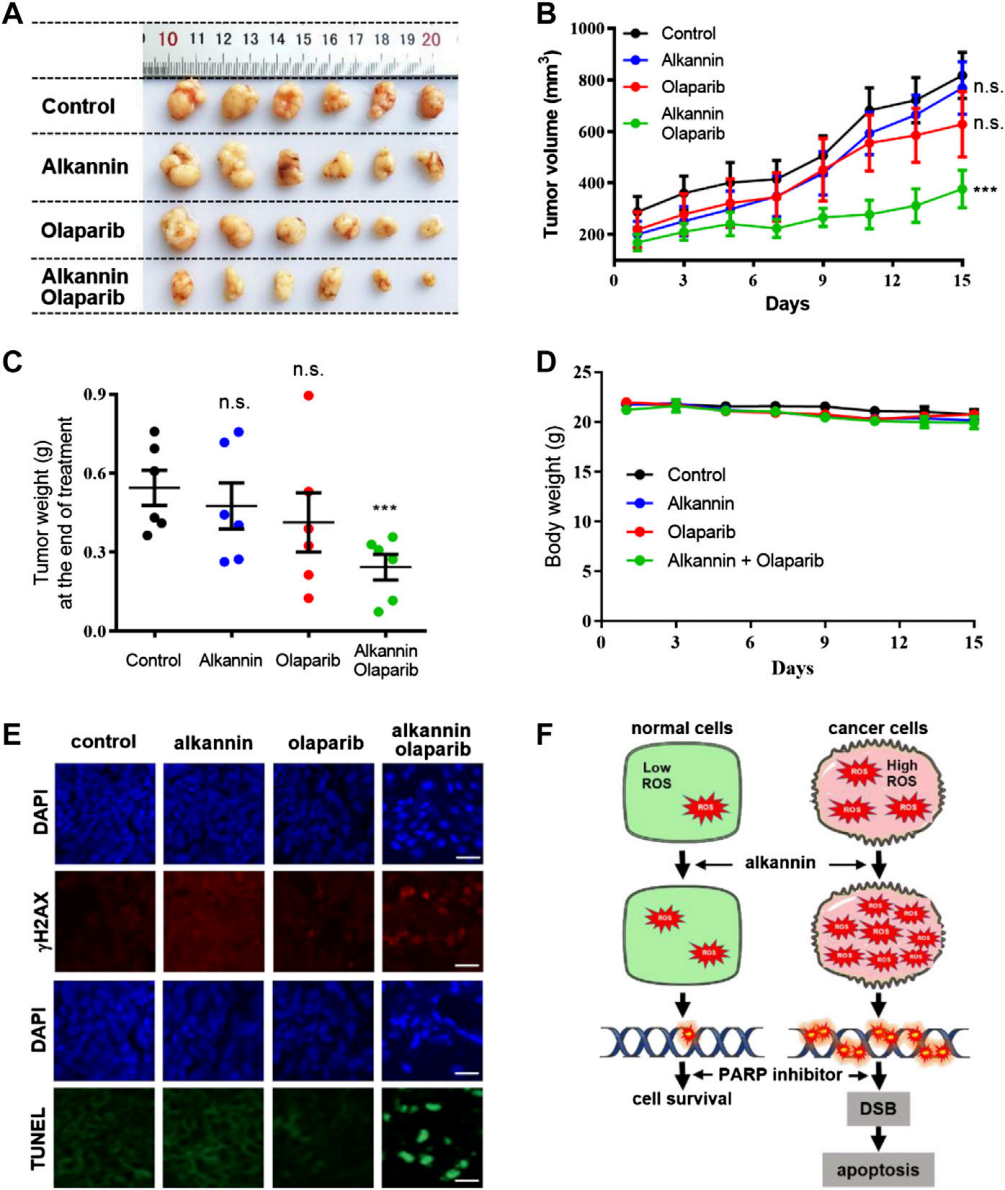
Alkannin and olaparib together suppresses growth of tumor xenografts *in vivo*. **(A)** Photograph of tumor mass dissected out at the time of study termination. **(B)** Tumor volume-time curve. Tumor volume was measured every 3 days and calculated according to formula *V* = 1/2 length × width^2^. **(C)** Tumor weight measured at the end of the study. **(D)** Body weight-time curve. Body weight was measured every 2 days. n.s. not significant, ***:*p* < 0.001. **(E)** Representative images of DNA damage (γH2AX) and apoptosis (TUNEL) in tumor tissues (scale bars: 20 μm). **(F)** Model for synergistic cytotoxicity of alkannin and PARP inhibitors in cancer cells.

To assess the clinical potential of the alkannin and olaparib combination, we evaluated the effects of treatment with alkannin and olaparib alone and combined on tumor xenografts *in vivo*. Mice bearing SW480 tumor xenografts were treated with either alkannin (dosed once daily by oral gavage, 20 mg/kg) or olaparib (dosed once daily by intraperitoneal injection, 50 mg/kg), or the combination of alkannin and olaparib, for 15 days. The results showed that alkannin or olaparib alone had no impact on the growth of the tumor xenografts, however, coadministration of alkannin and olaparib resulted in significant regression of the tumor xenografts (Figures 6A–C), demonstrating *in vivo* efficacy for the alkannin and olaparib combination. On the other hand, no significant difference in body weight was observed between control and the three drug-treated groups (Figure 6D), suggesting that the two drugs were well tolerated either alone or combined. Immunohistochemical staining of the tumor tissues revealed that only the group treated by the combination of alkannin and olaparib showed significantly increased γH2AX signals and intense apoptosis (Figure 6E), suggesting that DNA damage-induced apoptosis caused tumor regression.

## Discussion

Studies in the past have shown that PARP inhibition sensitizes cells to ionizing radiation and DNA damaging genotoxic agents irrespective of HR status (Bernges and Zeller, 1996; Trucco et al., 1998; Menissier de Murcia et al., 2003) and the activity of PARP1/2 in cancer cells is critical in the establishment of resistance to genotoxic radio- and chemotherapies (Sarkaria et al., 2008; Michels et al., 2013). These and many other observations all raise the awareness of the potential benefit of inhibiting PARP activity during radio- and chemotherapies, regardless of HR status. Many clinical trials have been carried out in the past decades to test the clinical use of PARP inhibitors in combination with radio- or chemotherapy. Regrettably, these clinical studies have ended with discouraging results due to normal tissue toxicity (Yap et al., 2019). Cancer cells typically exhibit high intrinsic oxidative pressure due to increased basal ROS output (Gorrini et al., 2013; AbdulSalam et al., 2016; Saikolappan et al., 2019). They are highly dependent on antioxidant systems to resist the toxicity of excessive levels of ROS, and hence are more sensitive to exogenous oxidative insult or inhibition of cellular antioxidant systems than normal cells (Trachootham et al., 2006; Gorrini et al., 2013; Harris et al., 2015; AbdulSalam et al., 2016). Accordingly, compounds that promote ROS generation or suppress cellular antioxidant systems have been found to cause ROS elevation and oxidative DNA damage selectively in tumor cells (Trachootham et al., 2006; Harris et al., 2015; Ding et al., 2016). In this study, we hypothesized that oxidative DNA damage selectively induced in cancer cells by pro-oxidative agents may synergize with PARP inhibitors to yield cancer-specific synergistic cytotoxicity.

Our results showed that nontoxic doses of alkannin induced a rapid increase in ROS levels in the SW480 and SW1116 colorectal cancer but not the NCM460 noncancerous colon epithelial cells. The enantiomeric alkannin and shikonin are known to be able to undergo cyclic oxidation and reduction (redox cycling) to generate ROS and deplete antioxidants (Wu et al., 2005; Kumagai et al., 2012; Yang et al., 2014; Qiu et al., 2018). As natural naphthoquinones, they also bind to and inhibit the activity of the thioredoxin reductase-1 (TrxR1) to cause ROS accumulation (Mau and Powis, 1990; Duan et al., 2014). The results of this study suggested that the antioxidant defense capacity of the SW480 and SW1116 cancer cells was exceeded by the combined effects of ROS overproduction and TrxR1 inhibition caused by the nontoxic alkannin, while the NCM460 colon epithelial cells was able to tolerate the effects due to normal basal ROS output. The sublethal alkannin-induced ROS increase in the cancer cells was followed by NAC-suppressible accumulation of 8-oxoG and DNA strand breaks including DSBs, indicating generation of oxidative DNA damage. Together, these results provide evidence showing that oxidative DNA damage can be induced selectively in cancer cells by nontoxic doses of pro-oxidative agents.

In agreement with that HR-proficient cells are insensitive to PARP inhibition, our study found that the SW480 and SW1116 cancer cells were highly resistant to the PARP inhibitor olaparib and veliparib. However, sublethal doses of alkannin strikingly sensitized the colorectal cancer cells to olaparib. The combination index (CI) values between sublethal doses of alkannin and olaparib indicated strong synergism. Thus, synergistic cytotoxicity in colorectal cancer cells was produced by the combination of alkannin and olaparib at nontoxic doses of both drugs. The synergistic cytotoxicity between alkannin and olaparib was greatly reduced by the ROS inhibitor NAC and by inhibition of OGG1, indicating that the synergy resulted primarily from base excision repair (BER) of oxidized DNA bases induced by sublethal alkannin. Furthermore, the combination of alkannin and the non-trapping PARP inhibitor veliparib did not yield synergistic cytotoxicity, suggesting that PARP-trapping at sites of SSBs, generated either directly by ROS or as intermediates of BER, was the major cellular event responsible for the synergistic cytotoxicity of the alkannin and olaparib combination.

Trapped PARP-DNA complexes combined with PARP inhibition-induced accumulation of unrepaired SSBs and impairment of replication fork stabilization and restart, induced replication stress and collapsed replication forks to promote generation of lethal DSBs (Hanzlikova and Caldecott, 2019). Our results showed that intense replication stress and increased DNA strand breaks including DSBs were induced specifically by the combination of alkannin and olaparib in colorectal cancer cells. Furthermore, both the ATM-Chk2 and ATR-Chk1 DDR signaling pathways were strongly activated, which were followed by profound activation of the G_2_/M checkpoint and the mitochondrial apoptosis pathway. Together, these results demonstrated that oxidative DNA damage selectively induced in colorectal cancer cells by sublethal alkannin synergized with olaparib-induced PARP inhibition and PARP-trapping to generate intense replication stress and extensive DNA strand breaks. The problems induced by the synergistic effects of sublethal alkannin and olaparib exceeded the repair capacity of the cancer cells, leading to DDR signaling to activate cell cycle checkpoints and the mitochondrial apoptosis pathway (Figure 6F). Some special agents, such as the NQO1 bioactivatable β-lapachone, the DNA methyltransferase-1 inhibitor guadecitabine, or a p53 activator APR-246, have been shown to increase oxidative pressure specifically in cancer cells, which also sensitized cancer cells to PARP inhibitors (Deben et al., 2016; Huang et al., 2016; Pulliam et al., 2018). Consistent with these findings, we observed that oxidative DNA damage induced by sublethal alkannin synergized with PARP inhibitors to yield cancer-specific cytotoxicity. Together, these studies support the exploration of synergistic cytotoxicity between PARP inhibitors and specific pro-oxidative agents to exploit a cancer vulnerability common to most tumor cells.

## Data Availability Statement

The original contributions presented in the study are included in the article/Supplementary Material, further inquiries can be directed to the corresponding authors.

## Ethics Statement

The animal study was reviewed and approved by The Institutional Animal Care and Use Committee of Jilin University.

## Author Contributions

MC, ZZ, and YS conceived the study. MC, HW, and JN performed the experiment, collected, and analyzed the data. MC, ZZ, and YS wrote the manuscript. All authors listed have made a substantial, direct, and intellectual contribution to the work, and approved the manuscript for publication.

## Funding

This work was supported by grants from the Natural Science Foundation of Jilin Province (20180101237JC) and Key Research and Development Program of Jilin Province (20170204025YY).

## Conflict of Interest

The authors declare that the research was conducted in the absence of any commercial or financial relationships that could be construed as a potential conflict of interest.
