# Supplementary material for: Alkannin-Induced Oxidative DNA Damage Synergizes With PARP Inhibition to Cause Cancer-Specific Cytotoxicity
Source: Front Pharmacol. 2020 Dec 22;11:610205. doi: 10.3389/fphar.2020.610205 (PMC7844861; doi:10.3389/fphar.2020.610205)
Supplement: Supplementary file 1 [file presentation1.pdf]

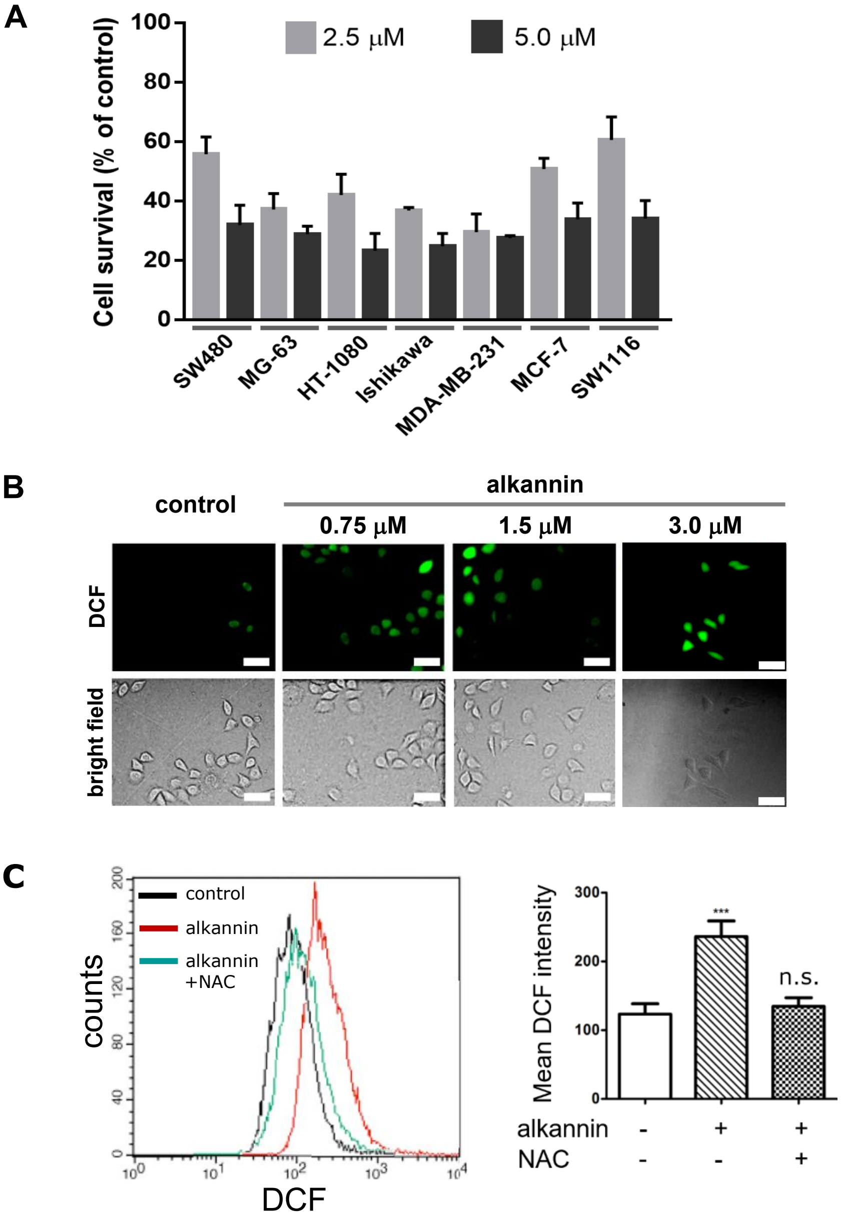

Figure S1. Sublethal alkannin elevates ROS levels in SW1116 colorectal cancer cells. **A** MTT assay. 24 h of treatment by alkannin dose-dependently inhibited the growth of the listed cancer cell lines (n = 3). **B** Representative images of SW1116 cells stained by DCFH-DA. 3 h of treatment by all three doses of alkannin induced a marked increase in ROS levels (scale bar: 50 μm). **C** Measurement of ROS by flow cytometry. 3 h of treatment by 0.75 μM alkannin induced a significant ROS increase in the SW1116 cancer cells, which was suppressed by NAC. Data of three independent experiments were presented as mean ± SD. n.s.: not significant, ***: $p < 0.001$ vs vehicle control.

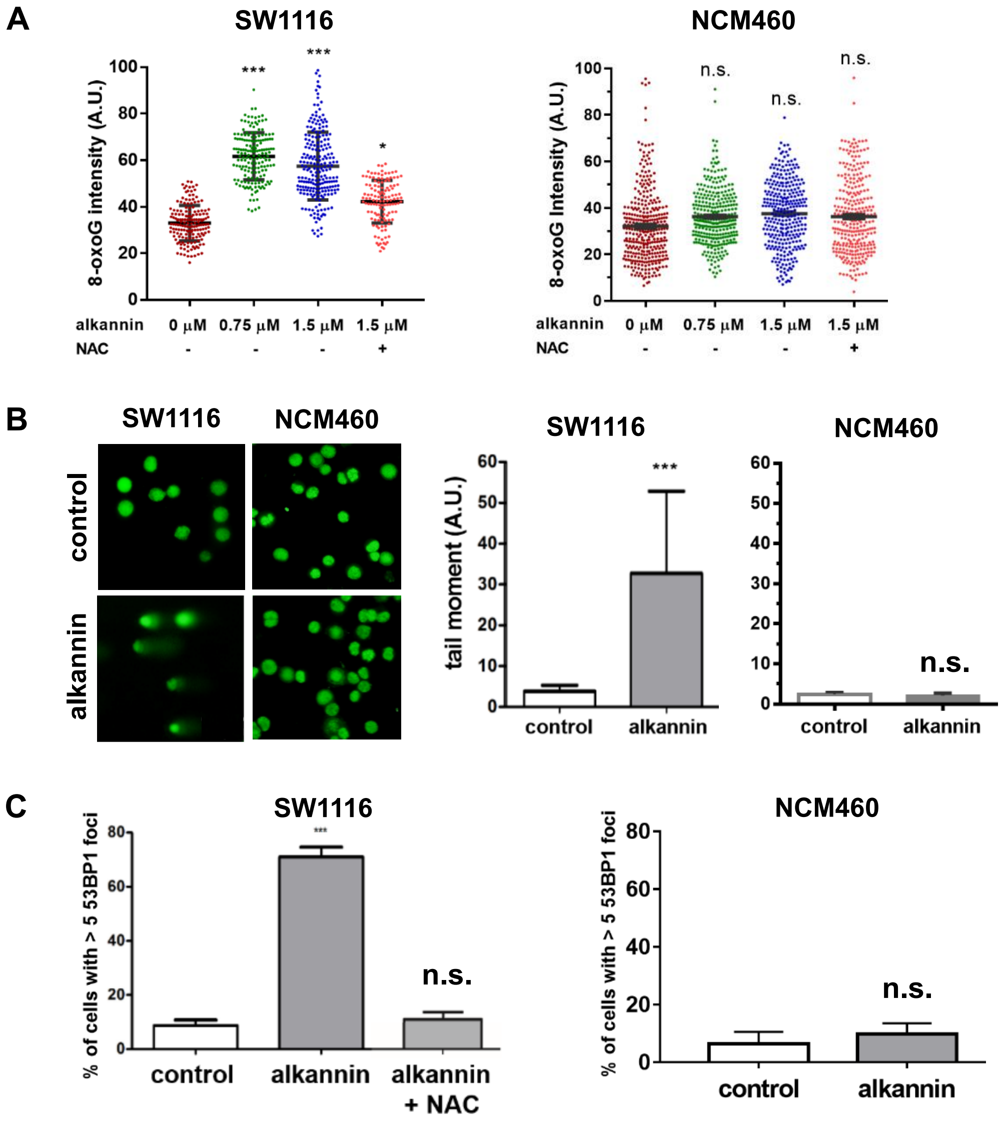

Figure S2. Alkannin-induced ROS elevation causes oxidative DNA damage in SW1116 cancer cells. **A** Measurement of 8-oxoG. 3 h of treatment by 1.5 and 0.75 µM alkannin induced a marked increase in 8-oxoG levels in the SW1116 cancer but not the NCM460 noncancerous cells (n = 3). NAC suppressed the 8-oxoG increase in the SW1116 cancer cells. **B** Alkaline comet assay. Treatment by 0.75 µM alkannin for 3 h significantly increased the numbers of DNA strand breaks in the SW1116 cancer but not the NCM460 noncancerous cells. Tail moment was quantified by the TriTek CometScore software (n = 3). **C** Induction of 53BP1 foci in SW1116 and NCM460 cells treated by 0.75 µM alkannin for 3 h (n = 3). n.s.: not significant, ***: $p < 0.001$ vs vehicle control.

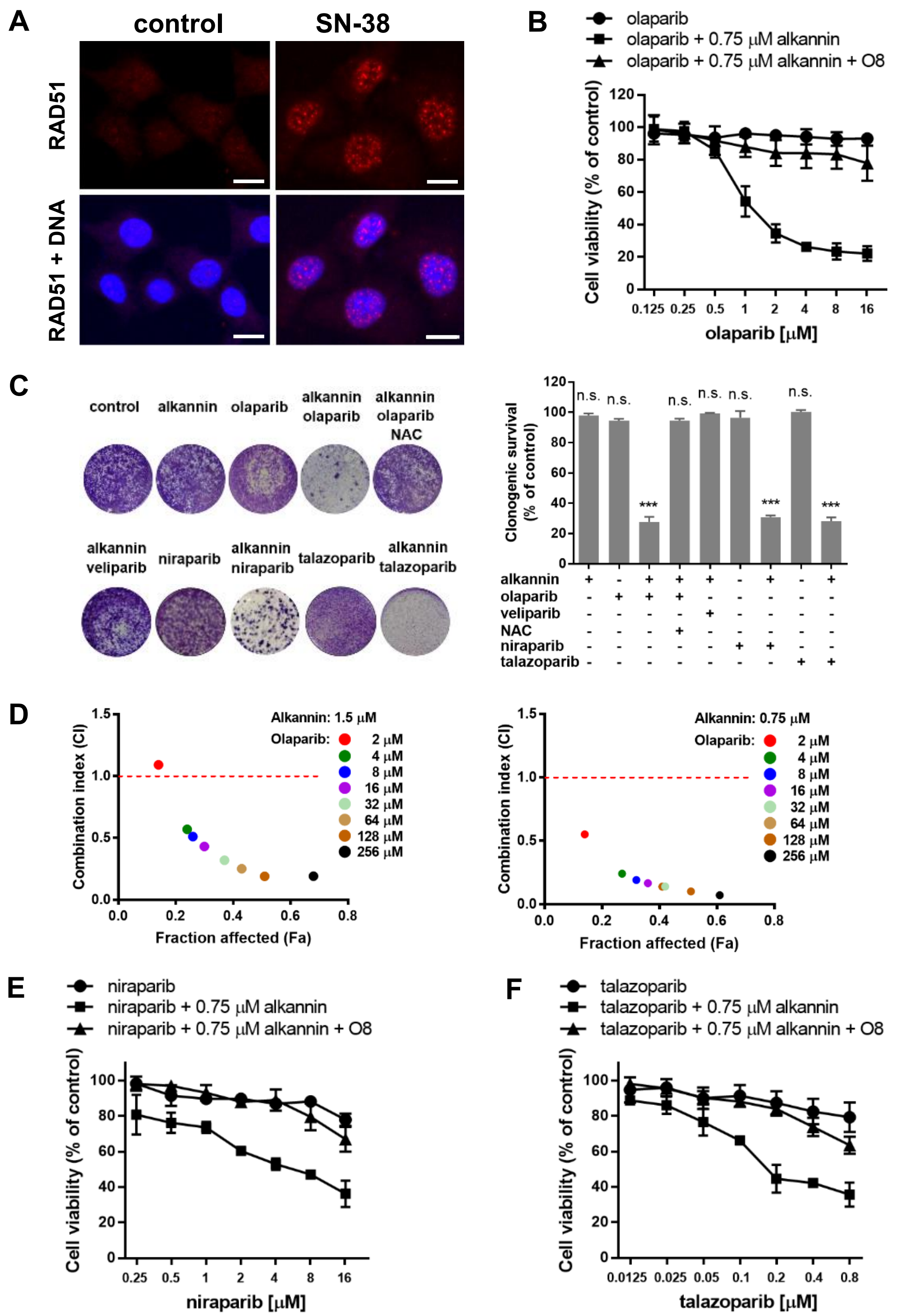

Figure S3. Sublethal alkannin sensitizes SW1116 cancer cells to PARP-trapping. **A** SW1116 cells readily formed RAD51 foci in response to 12 h of treatment by 5 μM SN-38 (scale bars: 10 μm). **B** MTT assay. SW1116 cells were treated with olaparib alone or together with 0.75 μM alkannin with or without the OGG1 inhibitor O8 for 72 h. **C** Colony formation assay. SW1116 cells were treated by the indicated drugs for 7 days (alkannin 0.75 μM, olaparib 10 μM, veliparib 10 μM, niraparib 5 μM, talazoparib 0.25 μM). Data from three independent experiments were presented as mean ± SD. **D** Determination of combination index (CI) values. SW1116 cells were treated by 1.5 or 0.75 μM alkannin and olaparib for 72 h. E-F: MTT assay. SW1116 cells were treated with niraparib (E) or talazoparib (F) alone or together with 0.75 μM alkannin with or without the OGG1 inhibitor O8 for 72 h. n.s.: not significant, ***: $p < 0.001$ vs vehicle control.

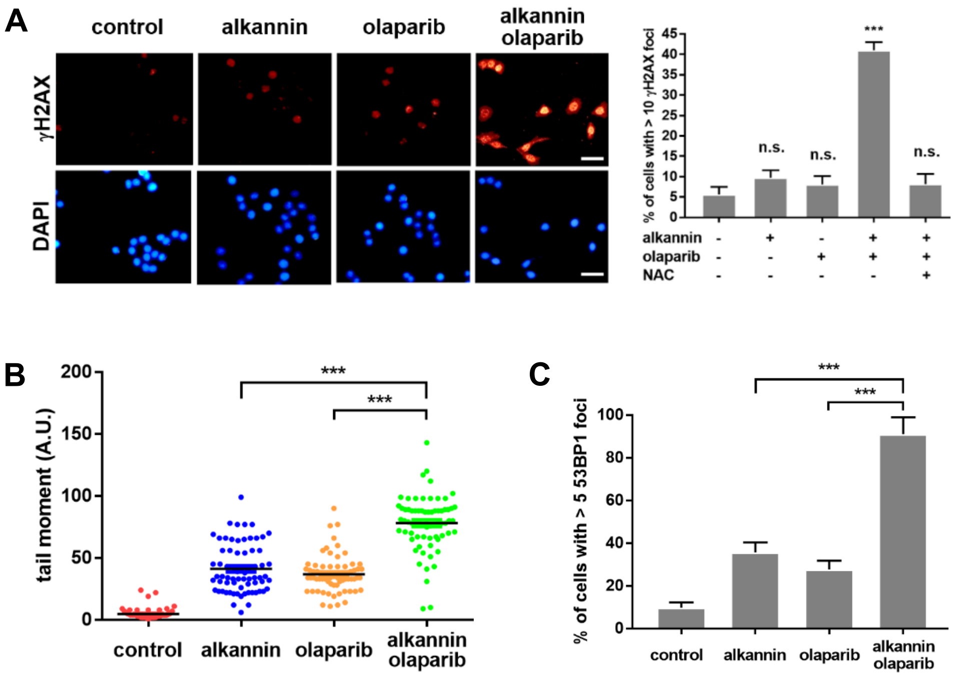

Figure S4. The combination of alkannin and olaparib induces intense replication stress and extensive DNA strand breaks in SW1116 cancer cells. SW1116 cells were treated by 0.75 μM alkannin, 10 μM olaparib or the combination of the two, with or without NAC, for 3 h. **A** Immunofluorescent staining of γH2AX (scale bars: 25 μm). Data from three independent experiments were presented as mean ± SD. n.s.: not significant, ***: $p < 0.001$ vs vehicle control. **B** Alkaline comet assay and **C** 53BP1 staining (n = 3). ***: $p < 0.001$, alkannin and olaparib combined vs alkannin or olaparib alone.

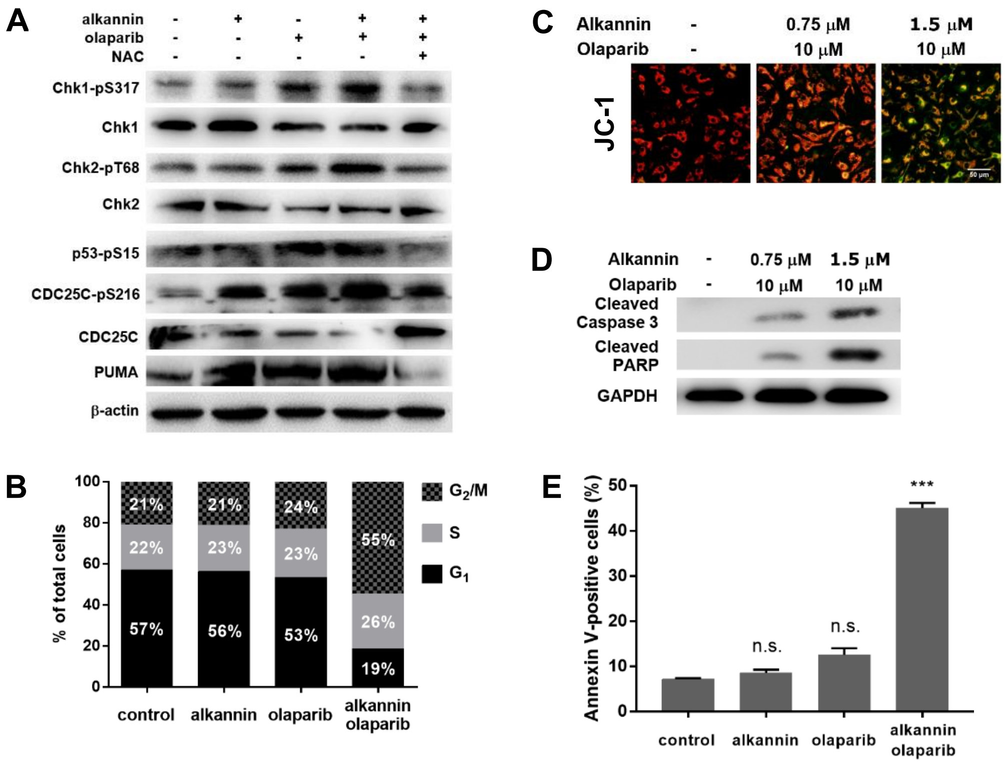

Figure S5. The combination of alkannin and olaparib activates DDR and induces cell cycle arrest and apoptosis in SW1116 cancer cells. **A** Western blot. SW1116 cells were treated by 0.75 μM alkannin, 10 μM olaparib or the combination of the two, with or without NAC, for 3 h. **B** Flow cytometry analysis of cell cycle. SW1116 cells were treated by 0.75 μM alkannin, 10 μM olaparib or the combination of the two for 48 h (n = 3). **C** Measurement of mitochondrial membrane potential and **D** Western blot. SW480 cells were treated by the combination of 0.75 or 1.5 μM alkannin and 10 μM olaparib for 12 h. **E** Flow cytometry analysis of apoptosis. SW1116 cells were treated by 0.75 μM alkannin, 10 μM olaparib or the combination of the two for 48 h (n = 3). n.s.: not significant, *: $p < 0.05$, ***: $p < 0.001$ vs vehicle control.
